# A Markov network approach for reproducing purchase behaviours observed in convenience stores

**DOI:** 10.1038/s41598-024-60752-w

**Published:** 2024-05-07

**Authors:** Dan Johansson, Hideki Takayasu, Misako Takayasu

**Affiliations:** 1 Department of Physics, Chalmers Institute of Technology, MSc. Complex Adaptive Systems, 412 96 Gothenburg, Sweden; 2 Department of Computer Science, School of Computing, Tokyo Institute of Technology, Yokohama, 226-8503 Japan; 3 Sony Computer Science Laboratories, Tokyo, 141-0022 Japan

**Keywords:** Complex networks, Computational science, Statistics

## Abstract

The convenience store industry in Japan holds immense significance, making a thorough comprehension of customer purchase behaviour invaluable for companies aiming to gain insights into their customer base. In this paper, we propose a novel application of a Markov network model to simulate purchases guided by stopping probabilities calculated from real data. Each node in the Markov network represents different product categories available for purchase. Additionally, we introduce the concept of a “driving force,” quantifying the influence of purchasing product A on the likelihood of purchasing product B, compared to random purchasing. For instance, our analysis reveals that the inclusion of nutrient bars in a purchase set leads to, on average, a 13% reduction in tobacco purchases compared to random patterns. To validate our approach, we compare the simulated macro-level purchase behaviours with real point of Sale (POS) data obtained from a prominent convenience store giant, 7-Eleven. The dataset is comprised of roughly 54 million receipts, in which we focus on the product categories existing in this dataset rather than individual products. Our model successfully replicates the purchase size distribution for 99.9% of all purchases and the purchase counts across various product categories, demonstrating its efficacy in capturing broad purchase patterns.

## Introduction

Convenience stores are an integral part of Japanese daily life, offering a wide range of essential items, which contributes to their immense popularity. This paper presents a model that effectively captures customer behaviour by modelling point of sale (POS) data from convenience stores. POS data comprises crucial information recorded during a purchase, including the purchased products, time, location, and payment method. The model to be proposed is based on POS data obtained from 7-Eleven, the largest convenience store brand in Japan, with over 21,000 stores and gross sales figure of more than 5 trillion Yen (33.7 billion USD)^1^. Given the significant size and substantial financial transactions within the convenience store industry, there is a strong incentive to comprehend customer behaviour accurately.

In this paper, a novel approach to simulate purchase behaviours using a Markov model is proposed, which incorporates innovative techniques for both the selection of the starting state and the stopping of the Markov process through the introduction of stopping probabilities. The versatility and well-established nature of the Markov process have led to its widespread utilization in various domains, including medicine^2^^,^^3^, chemistry^4^, economics^5^, engineering^6^, network optimisation^6^, and disease modelling^7^.

The primary objective of our research is twofold: firstly, we aim to investigate whether a simple probabilistic model like the Markov model can effectively replicate the complex purchase dynamics observed in real purchase data. Secondly, we introduce the concept of a purchase “driving force,” which quantifies the influence of purchasing product B on the likelihood of subsequently purchasing product A, as opposed to making random purchases. In essence, this metric measures the inclination to buy more items from category A after already choosing a product from category B.

We introduce this concept with dual motivations: first, it provides an intuitive and quantitative means of assessing conditional purchase probabilities between product categories. Secondly, by confirming the existence of these driving forces within actual purchase data, we can investigate the model’s ability to capture and replicate this behaviour in a meaningful and statistically significant manner. While the exploration of conditional probabilities in consumer purchase behaviour is not novel, traditional methods like bivariate hazard modelling^8^ and Bayesian modelling^9^ have been applied in previous studies to model conditional probabilities. We believe that our definition of driving force and its components present a novel approach to model conditional probabilities within a system, and provides future research within this field with an additional tool to understand these behaviours.

While the implementation of the Markov model presented in this paper to model purchase behaviours within convenience stores may be innovative, the use of Markov processes to model human behaviours is not new. Previous studies have employed Markov models to explore population mobility behaviour^10^, web-browsing behaviour^11^, traffic^12^ and driving behaviour^13^ and e-commerce purchase behaviour^14^ among other examples. Despite its prior applications in different contexts, we believe that a model that can capture the purchase behaviours observed in connivance stores using a Markov process, and the concept of driving force are novel and important additions to the study of purchase behaviour modelling.

The key finding of this paper is that the Markov models developed in this study effectively replicated purchase patterns seen in the POS data. These simulated purchases successfully captured various purchase behaviours, including purchase size and accurate distribution across product categories. It was also found through big data analysis that the purchase size distribution has a power law tail which cannot be explained by a Markov chain model. By introducing an additional non-Markovian effect to the model depending on the purchase size, the power law tail distribution is realised. Furthermore, our analysis confirmed the existence of driving forces influencing purchasing decisions between different product categories in real-world data. While the Markov model demonstrated the capacity to capture this behaviour to some extent, it did not fully replicate the complexity of these inter-category influences.

The same dataset used for the making of this model have also been used in previous studies to better understand the dynamics in connivance store data, such as the metabolism of products over time^15^, optimisation of product inventory for reduced waste and higher profit^16^, estimation of change of probability for purchase of commodities using Possion process^17^ and power law relationships of product sale fluctuations^18^.

### Limitations

There are a couple of noteworthy limitations in this study. Firstly, the POS data is treated as static and uniform, without accounting for temporal fluctuations, such as seasonal variations, time of year, or time of day. Secondly, the primary focus of this study centres on the transition between category states within the Markov model, specifically the inter-category relationships. As a result, the analysis and methodology in this paper are confined to purchases of size 2 or larger.

## Results

**Figure 1:**
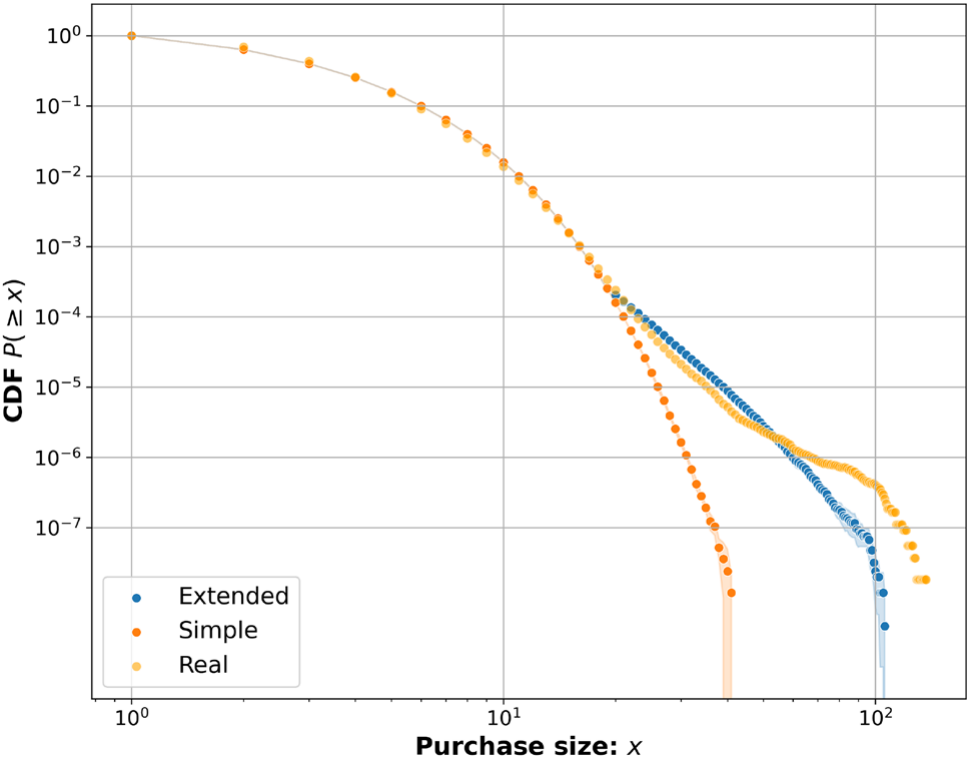
The plot illustrates the purchase size distribution in three different models: real data, simple Markov model and extended Markov model and their respective mean and 95% confidence intervals.

Using two different Markov models (Methods—Markov purchase model) $5*10^7$ simulated purchases were generated using each model, matching the number of purchases in the real data. Figure 1 displays the cumulative distribution of the purchase size for both the real data and the two implemented versions of the Markov model: the simple model and the extended model. The simulation was run 10 times for both the simple and extended model, the resulting mean is represented by the points and the coloured area is the 95% confidence interval for each model. We observe that the confidence interval is narrow except for when the purchase size is very large, this shows that the model results are very consistent between runs.

Notably, the simple Markov model closely aligns with the real data for purchase sizes up to 18, recreating more than 99.9% of the real purchase sizes before diverging. However, it fails to replicate the power law tail observed in the real data for large purchase sizes. To address this limitation and better capture the behaviour of larger purchases, the extended Markov model was introduced. In this model, the simple Markov model’s stopping probability $P_S(c)$ (Eq. 2) is used for purchase sizes up to 18 products. For purchases larger than 18, a modified stopping probability $P_{S,t}(c,n_t)$ (Eq. 3) is utilised. As depicted in Fig. 1, the extended model proves to be more successful in capturing the power law tail observed in the real data for large purchases. This shows the extended model’s ability to accurately reproduce the real data’s behaviour for both small and large purchase sizes.

**Figure 2:**
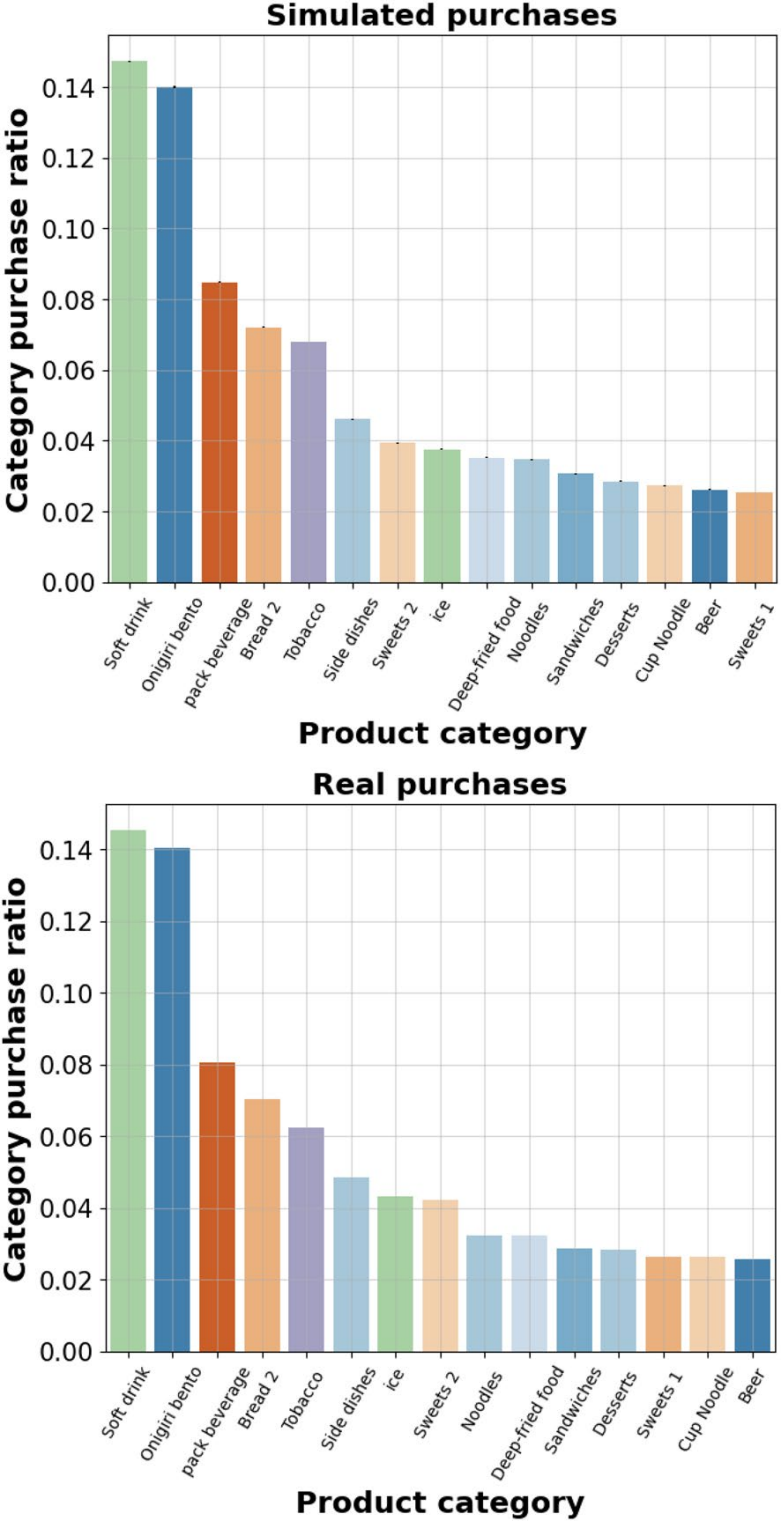
Purchase ratio ranking comparison for real purchases and extended Markov model simulated purchases.

Shifting focus to the product category purchase ratios. Figure 2 displays a comparison of the purchase ratio for the top 15 product categories for the real data and extended Markov model. All categories and their ranking are shown in Table 1. Observe that the order of magnitude is consistent between the model and real data and the ranking of the categories are mostly consistent. Notable ranking divergences between the simulated purchases and the real data, are categories “Ice” and “Sweets 2” , “Noodles” and “Deep-fried food” and finally “Cup Noodle”, “Beer” and “Sweets 1” are inconsistent. The standard deviation in the simulated data is very small, barely showing up as tiny black dots on top of the category bars in the simulated data, this would indicate that the inconsistency in the ranking is not the result of randomness but due to some systematic error. It is also worth mentioning that all category ranking inconsistencies are from categories which are very close in purchase ratio magnitude. These results suggests that the extended Markov model is able to capture the macro behaviours of the real data, i.e. purchase size distribution and the category purchase ratio, to a great extent.

**Figure 3:**
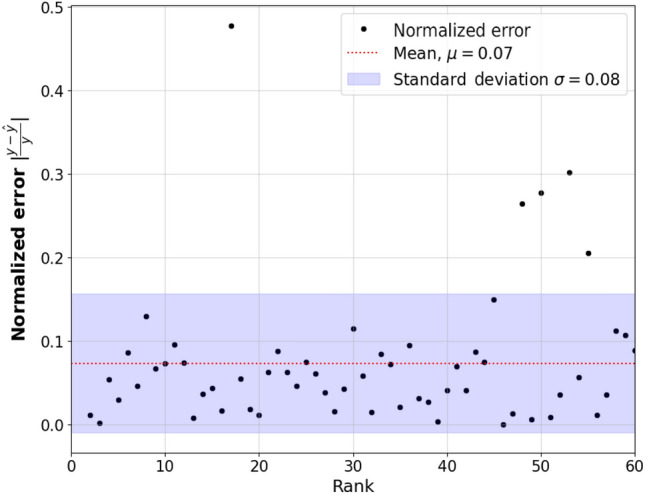
Absolute normalised error for the purchase ratio of all categories ordered according to their purchase ratio rank from Table 1.

To assess the accuracy of the purchase ratio results, the normalised absolute error ($\text {NAE} = \left| \frac{y-\hat{y}}{y}\right|$) was calculated for each category, where *y* is the real data and $\hat{y}$ is the results from the extended model. Utilizing this result, the mean absolute percentage error ($\text {MAPE } = \frac{100}{C}\sum _{k=1}^C \left| \frac{y-\hat{y}}{y} \right|$) was also computed, which was found to be 7.36%. Figure 3 shows the calculated errors for each category, the errors seem to be non-systematic, and the majority of them lie within one standard deviation. However, there are a few significant outliers that warrant attention. The rank 16 category “Oden” (Japanese stew), which is typically sold during the winter season, exhibits a larger error. This behaviour might be attributed to the seasonal nature of the product, requiring extra care in the modelling process to accurately capture its sales patterns. Rank 47 Category “Copy” poses challenges in modelling due to customers paying directly at the copy machine instead of at the cashier, as a result, this category’s behaviour might not fully align with the Markov model, contributing to the larger error. Similarly, rank 49 “Cash voucher”, rank 52 “Store gift” and rank 54 “Game software” also present outliers in the error plot. The nature of these categories, involving non-typical purchase patterns, could contribute to the discrepancies between the real data and the model’s predictions. It is noteworthy that the larger errors tend to occur for lower-ranking categories or those with unique purchase behaviours, like seasonal products or specialised items. These nuances and outliers should be taken into account when interpreting the results and making model-based predictions.

### Driving force

**Figure 4:**
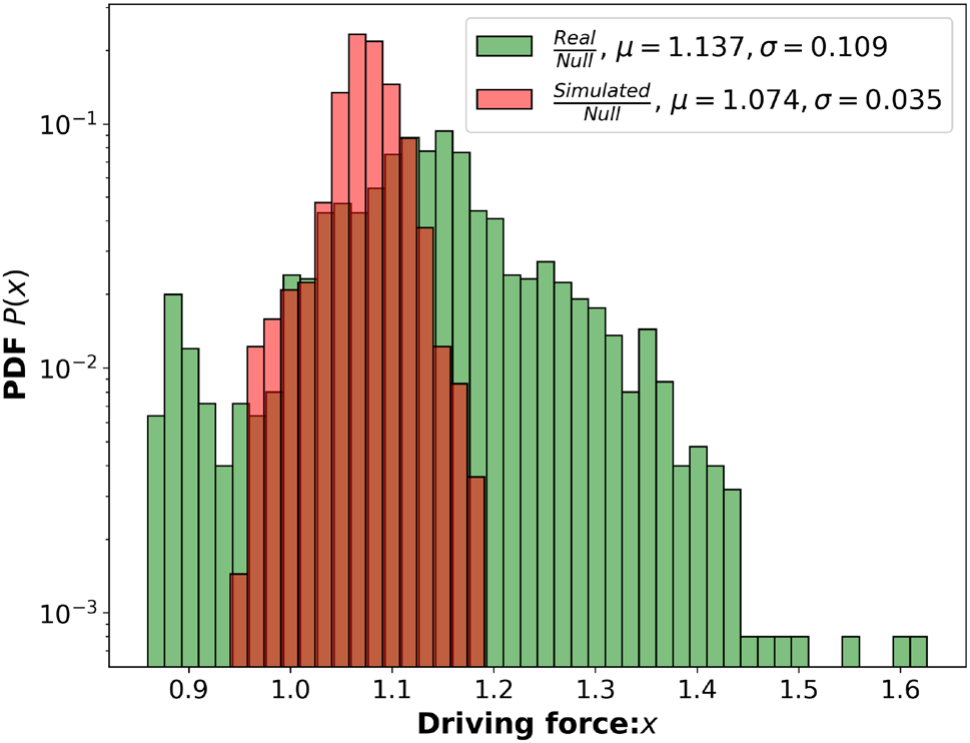
Driving Force Histogram. The histogram presents three driving force distributions: in green, the PDF for Real-Null driving force; in red, the driving force distribution for Simulated-Null.

The driving force ratios (Methods - Driving force) was calculated for three combinations of purchase data (Eq. 5). These combination were: real data—null model and simulated data—null model, with the null model representing complete random purchasing. These results can be seen in Fig. 4, in which the three distributions seem to be distinct from one another, providing evidence of the existence of driving forces in the real data. However, it seems also that the model fails to capture the specific driving forces observed in the real data. A few intuitive examples of driving forces observed in the real data are

- Soft drink $\rightarrow$ ice: 1.25
- Tobacco $$\rightarrow $$ Beer: 1.12
- Nutrient bars $\rightarrow$ Tobacco: 0.87

These ratios imply that, for instance, if soft drinks are selected for purchase, there is a 25% increase in the likelihood of purchasing ice compared to a random selection. Conversely, the purchase of nutrient bars is associated with a 13% decrease in the likelihood of buying tobacco products. To statistically validate the existence of driving forces in the real data, a two sample Kolmogorov-Smirnov (KS) test was conducted. This test compared the distribution of the driving forces in the real and simulated data. The test resulted in a KS statistic of 0.495 and a p-value of $2.29\times 10^{-147}$, indicating significant differences between these distributions. This outcome confirms the presence of driving forces in the real data. However, it also highlights the model’s limitation in fully capturing these forces.

## Discussion

As observed in the results shown in Fig. 1, the simple Markov model demonstrated high accuracy in modelling approximately 99.9% of the purchase size cumulative distribution function (CDF). The remaining 0.1% of larger purchases, which could be considered outliers, might not be crucial to model accurately. This raises the question of whether there is a real need for the extended Markov model. In the context of this specific dataset, achieving 100% accuracy in modelling all purchases might not be essential. Instead, the focus could be on accurately modelling the more common purchase sizes. However, both the simple Markov model and the extended Markov model remain valuable contributions, especially for other potential datasets where the purchase distribution could differ significantly. The extended model’s ability to introduce a power law tail proves valuable in such scenarios. Regarding the purchase ratio results, there were some outliers with larger errors. For instance, rank 16 category “Oden” (Japanese stew), being a seasonal product, exhibits a unique purchase behaviour that is not fully captured by the static model used in this study. Introducing a time-dependent transition matrix would be a natural next step to address this limitation. By incorporating temporal dynamics, the model could better represent seasonal or daily purchase behaviours. Additionally, a significant weakness of the current dataset is the lack of information about the purchase order of products. In the model, this is approximated using the starting node scheme (Eq. 4). However, there is currently no way to measure the accuracy of this approximation, and the starting node probabilities are heavily influenced by the transition probabilities (Eq. 1). In reality, the first product selected, which drives the purchase, may have a more nuanced effect on subsequent purchase decisions. Developing methods to better infer and incorporate the driving product category could significantly enhance the model’s accuracy. Moreover, the model could be improved to more accurately capture single-item purchases. In such cases, the driving product is known since it is a single item. Calculating the purchase ratio specifically for single-item purchases and then performing a weighted combination with the purchase ratio for purchases larger then 1 could lead to a more comprehensive and accurate representation of customer purchase behaviours.

The concept of the driving force in real data was intriguing, and while it seems to exist intuitively, proving its presence was an exciting result. However, as stated in the results, the model struggled to fully capture these micro-level purchase behaviours. Complex systems often exhibit simpler macro behaviours that are easier to model, whereas the micro behaviours, such as individual human purchase decisions, tend to be more challenging to represent probabilistically, especially within a Markov model framework. Human purchase behaviour is inherently non-probabilistic, as customers typically enter a store with a predetermined agenda, which poses a significant challenge for probabilistic models like the Markov model. To improve the model’s ability to capture micro-level behaviours, a potential avenue for future research involves implementing a weighting scheme for the transition probabilities using the driving force, thus letting already selected products influence the future transitions. This introduces another form of memory into the model, further deviating it from being purely Markovian. However, this approach could potentially lead to a more accurate representation of human purchase behaviour by better considering the influence of past purchases on current decisions.

## Methods

The method section will contain three major subsection, firstly an analysis of the POS data to give key insight into the purchase behaviours and data distributions, which leads into the second subsection on constructing the Markov purchase model. The section then ends with the introduction and definition of driving force.

### Data analysis

In accordance with the relevant guidelines and regulations outlined in the editorial and publishing policies of *Scientific Reports*, all methods employed in this study were conducted with strict adherence to established standards. The experimental protocols employed in this research were ethically sound and received approval by Misako Takayasu Lab. which are responsible for overseeing these research activities. Furthermore, the dataset used for this research was completely anonymised before acquisition of Misako Takayasu Lab. We affirm that informed consent of the affected parties, as required by the guidelines and regulations was upheld.

The data analysed and used to develop the purchase simulation model comes from 7-Eleven point of sales data (POS). POS data is data collected during a customer sales transaction and usually contains a large set of features such as time of purchase, cost metrics and product categories. Since POS data is usually feature rich, it may require a lot of preprocessing to be used in a model. The POS data was collected from 326 chain stores of the leading Japanese convenience store company, 7-Eleven Japan Co., Ltd., during 153 days from 1 June to 31 October 2010. More specifically, the data was sourced from 326 stores located in Kanagawa prefecture and Yamaguchi prefecture. Individual owners mainly franchise the stores and sell an extensive range of products, such as processed food, fast food, daily perishables, and non-food items. In total the dataset contains 60 unique product categories, these categories will be the focus of the paper rather than the individual products within these categories. The receipt dataset that was used for the analysis contains 54,099,713 unique customer purchases during this time period. All categories present in the data including their rank, purchase count and purchase ratio can be found in Table 1, which shows that the distribution of category popularity follows a significant disparity. The top-ranking categories have immense popularity, being orders of magnitude more frequented than the lower-ranking ones. Notably, the purchase count experiences a rapid decay as the ranking increases. The data reveals a clear concentration of purchasing activity around a few highly favoured categories, while a vast array of lower-ranking categories experiences comparatively lower demand.

**Table 1:** Category rank table showing all categories in the network and their ranking according to their respective purchase counts.

Rank	Category name	Purchase count	Purchase ratio	Rank	Category name	Purchase count	Purchase ratio
1	Soft drink	19,382,223	0.145518531	31	Hair styling products	402,907	0.003024959
2	Onigiri & Bento (Lunch box)	18,699,674	0.140394066	32	Manga books	348,149	0.002613845
3	Pack beverage	10,715,945	0.080453546	33	Office supplies	320,661	0.00240747
4	Bread 2	9,349,197	0.070192228	34	Nutrient solid food	310,624	0.002332114
5	Tobacco	8,323,758	0.062493401	35	Seasoning	254,929	0.001913965
6	Side dishes	6,439,963	0.048350179	36	Tissues	221,133	0.00166023
7	Ice	5,754,022	0.043200247	37	Cheese	214,416	0.0016098
8	Sweets 2	5,598,961	0.042036075	38	Food products	200,819	0.001507716
9	Noodles	4,293,192	0.032232577	39	Cleaning supplies	192,273	0.001443554
10	Deep-fried food	4,278,475	0.032122084	40	Assortment etc.	168,545	0.001265408
11	Sandwiches	3,814,734	0.028640393	41	Egg	167,240	0.00125561
12	Desserts	3,759,675	0.028227019	42	Detergent	165,953	0.001245948
13	Sweets 1	3,506,748	0.026328085	43	Bath & Oral care	159,524	0.00119768
14	Cup Noodle	3,483,621	0.026154451	44	Stamp	124,367	0.000933727
15	Beer	3,397,907	0.025510925	45	Men’s clothing	97,526	0.000732209
16	Oden (Japanese stew)	2,764,175	0.02075297	46	Medical supplies	95,418	0.000716383
17	Magazines	219,9692	0.016514924	47	Copy	79,007	0.000593172
18	Snacks	1,899,244	0.01425921	48	Paperback	47,069	0.000353386
19	Energy drinks	1,712,964	0.012860651	49	Cash vouchers	40,041	0.000300621
20	Bread 1	1,663,500	0.012489283	50	Coffee (Self service)	18,791	0.00014108
21	Newspapers	1,663,499	0.012489276	51	Rice	13,582	0.000101971
22	Chuhai plum wine	1,524,602	0.01144646	52	Store gift	10,435	7.83E-05
23	Snacks	1065922	0.008002766	53	Movie DVDs etc.	1983	1.49E-05
24	Frozen food	991,286	0.007442412	54	Game software	1344	1.01E-05
25	Packed food	812,059	0.006096805	55	Boxed products	1254	9.41E-06
26	Electrical system	570,731	0.004284954	56	Electronic products & Calendars	413	3.10E–06
27	Jelly cake mix tea bags	539,344	0.004049306	57	CD etc.	364	2.73E–06
28	Sake shochu (Alcohol)	478,990	0.003596178	58	Promotion	221	1.66E–06
29	Character product	430,491	0.003232055	59	For tasting	164	1.23E–06
30	Handmade products	420,443	0.003156617	60	Coupon	2	1.50E–08

The table also contains the purchase ratio of each category which is the purchase count divided by the sum off all purchase counts.

**Figure 5:**
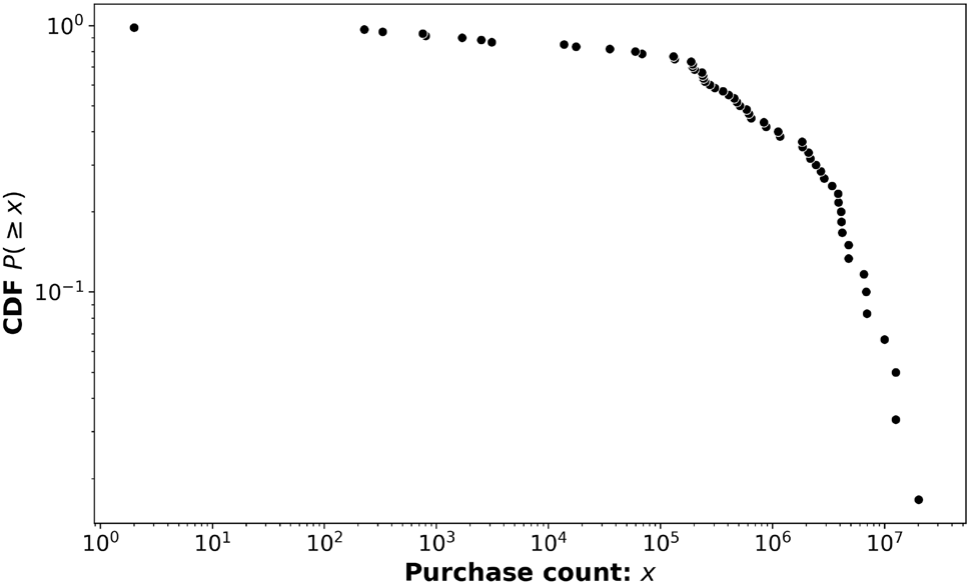
Cumulative distribution of purchase count for all product categories.

In Fig. 5, the CDF of product category purchase counts is observed. Notably, there is a region of slow decay in the CDF for purchase counts ranging from 1 to $10^5$, followed by a rapid decay as the purchase count increases towards the maximum of approximately $10^7$. This pattern suggests that there are numerous product categories with a relatively moderate purchase frequency, but only a few categories with extremely high purchase counts. The significance of this observation warrants further investigation.

**Figure 6:**
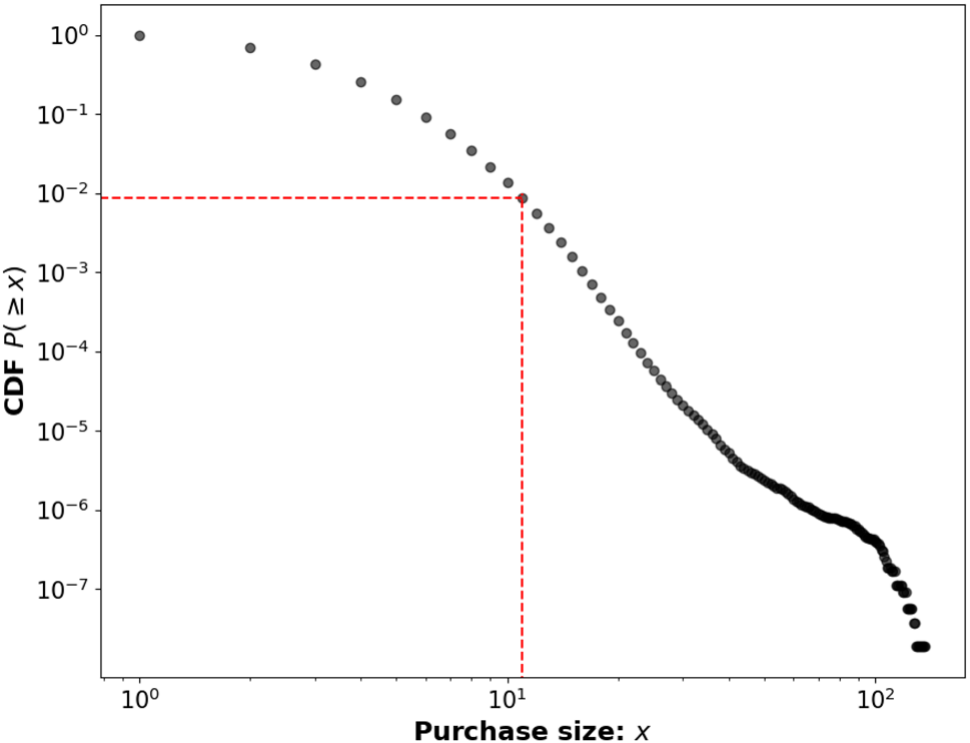
Cumulative distribution of purchase size distribution. The red dotted lines highlight where the CDF reaches a value of $\sim 10^{-2}$ at a purchase size of 11, representing that a purchase size of 11 includes 99% of all purchases.

Another essential purchase behaviour to explore is the purchase size distribution, which reflects the number of items people tend to purchase. Figure 6 shows the cumulative distribution for purchase sizes. Notably, around 40% of all purchases consist of single-item purchases. Additionally, the red dotted line indicates that 99% of all purchases comprise of 11 items or fewer. We can further discern that in the 1–10 item range, in a log-log plot the CDF decays exponentially, whereas for purchases larger than 10 items, the CDF exhibits a more linear in behaviour. This suggests the existence of a power law like relationship for larger purchase sizes, which is a crucial aspect to consider when developing the model later on. Understanding the distribution of purchase sizes provides valuable insights into the typical buying habits of the population under study.

The category matrix $\textbf{M}$ is a $C \times C$ matrix, where each element $m_{i,j}$ represents the number of times category *i* was purchased together with category *j*. In the case of this dataset since it contains 60 categories, *C* is equal to 60. This matrix serves as a fundamental component in our investigation of product category relationships. To construct $\textbf{M}$, we calculated the co-occurrence counts using all available purchase data. A subset of the resulting matrix is showcased in Table 2, providing concrete values for specific example categories. A heat map showing all categories and their respective number co-occurrences can be seen in Appendix 5 Fig. 10. It is worth emphasising that certain combinations of categories were never purchased together, resulting in zero entries in $\textbf{M}$. Notice also that ${\textbf {M}}$ is symmetric.

**Table 2:** Subset of category matrix $\textbf{M}$ containing the purchase count for all possible combinations of categories in the data.

	Soft drink	Beer	Sweets	$\ldots$
**Soft drink**	2,142,460	189,494	425,541	$\ldots$
**Beer**	189,494	117,951	81,364	$\ldots$
**Sweets**	425,541	81,364	222,048	$\ldots$
$\vdots$	$\vdots$	$\vdots$	$\vdots$	$\ddots$

The inherent nature of the category matrix introduces a constraint as it exclusively considers purchases with a purchase size *n* greater than 1. Moreover, the concept of ’driving forces’ between products is meaningful only within the context of purchase sizes where $n > 1$. Consequently, this paper, along with the proposed methods and results, is intentionally confined to an analysis focusing solely on purchases with a purchase size greater than 1 which effectively eliminates roughly 40% of the purchase data. We found that single item purchases behave differently than multi item purchases since there is no inter-category relationships present.

### Markov purchase model

The Markov model aims to generate synthetic purchase data for comparison with actual purchase data. This model comprises three key components. Firstly, the starting node selection determines the initial product category from which the simulated purchase process begins. This choice is crucial, given that most purchases typically involve only a few items. Secondly, the transition probabilities govern the movement between different states within the state space, where each state represents a product category available for purchase. Lastly, a stopping probability is introduced to determine when the purchase process concludes.

To gain insight into the network structure of the model, let us see how the transition probabilities between nodes were calculated. The transition matrix ${\textbf {P}}$, also a $C \times C$ matrix, is derived from the category matrix ${\textbf {M}}$. The rows of ${\textbf {P}}$ represent source nodes, while the columns represent destination nodes. The elements in ${\textbf {P}}$ correspond to the transition probabilities between nodes in the network and are defined as follows for a given transition from node j to i.

1 $$\begin{aligned} P_T(i|j) = \frac{m_{j,i}}{\sum ^C_{k=1} m_{j,k}} \end{aligned}$$

Equation (1) normalises each value in ${\textbf {M}}$ by dividing it by the sum of its respective row, yielding the transition probabilities. The sum of each row in ${\textbf {M}}$ represents the count of how many times a category was involved in any purchase combination. Since these sums are unique for each category, the symmetry of ${\textbf {M}}$ is lost during the calculation of ${\textbf {P}}$, resulting in non-symmetric transition probabilities between nodes.Importantly, both ${\textbf {M}}$ and ${\textbf {P}}$ contain zero values due to the absence of certain category combinations in the purchase data. Consequently, the network represented by ${\textbf {P}}$ is not fully connected.

**Figure 7:**
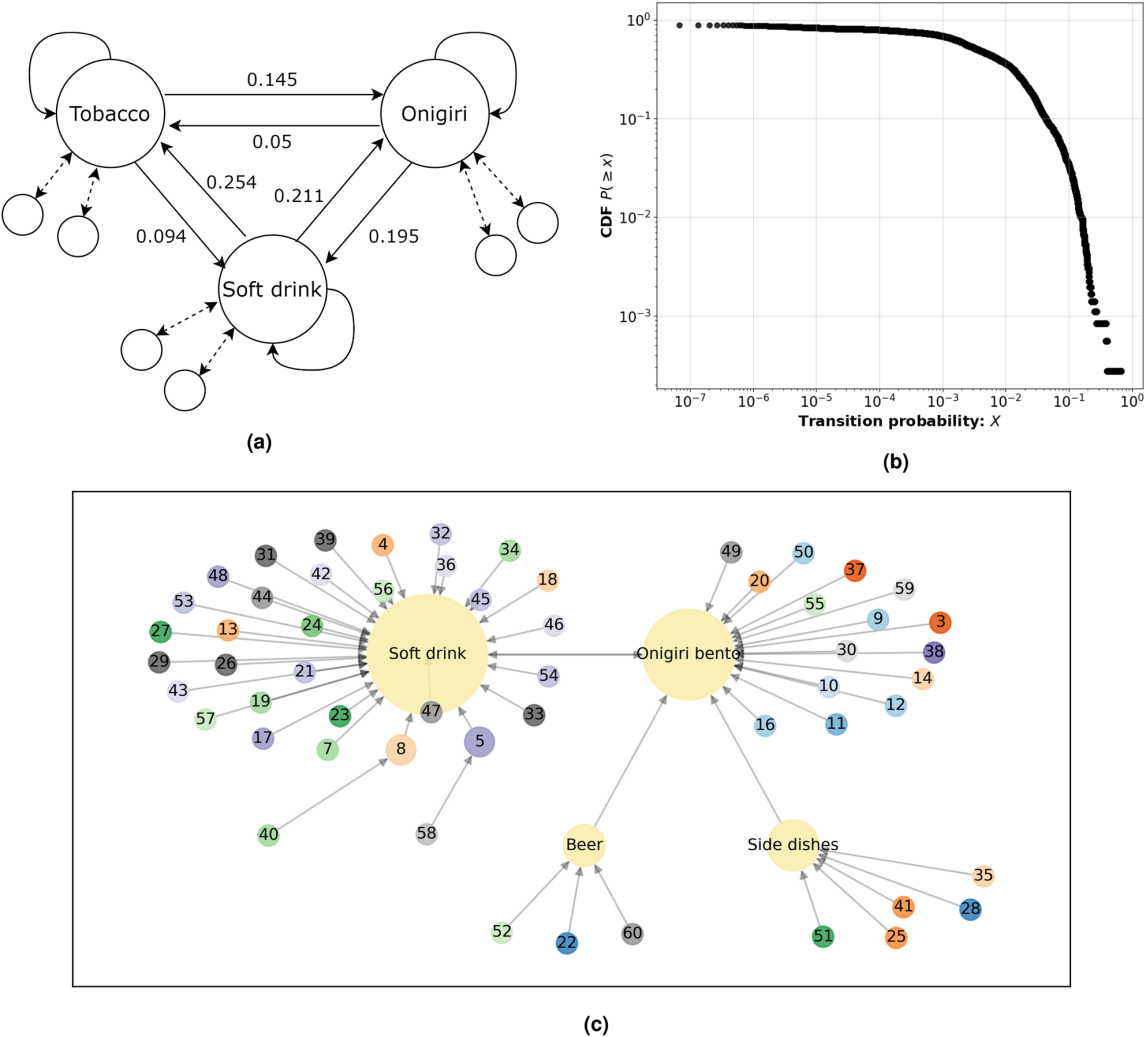
(**a**) Sub-graph example of category network network showing some popular categories and their respective transition probabilities. (**b**) Cumulative Distribution of all transition probabilities in network. (**c**) Category network with largest transition probabilities and node sizes reflecting connection frequency, the numbered nodes are represented by their rank. The figure displays a category network representing transition probabilities between all nodes (excluding self-connections). Node sizes are proportional to the number of edges connecting to each category. Significant nodes include ’Soft Drink,’ ’Onigiri/Bento,’ ’Side Dishes,’ and ’Beer,’ which exhibit a higher number of connections (above 2).

Figure 7a illustrates an example sub-graph of the category network, displaying transition probabilities between a few selected nodes. Notably, the transition probabilities are non-symmetric, and self-connections are present, representing instances of buying multiple items of the same category in sequence. Additionally, the non-fully connected nature of the network is shown.

The cumulative distribution of the transition probabilities in ${\textbf {P}}$ is illustrated in Fig. 7b. The figure provides us with a better understanding of the nature of the transition probabilities within the network, notably the CDF exhibits two distinct regions. In the first region, where the transition probabilities are $< 10^{-3}$, a gradual decline in the CDF value is observed, indicating an abundance of transition probabilities in this range. This implies that the majority of connections between nodes are characterised by relatively weak transition probabilities. Conversely, in the second region, where the transition probabilities are $> 10^{-3}$, the CDF shows a rapid decrease. This suggests that larger transition probabilities are infrequent within this network. In essence, strong connections with high transition probabilities are rare occurrences. To gain a deeper insight into the network structure, a network graph plot was constructed and is presented in Fig. 7c. In this visualisation, each node is represented by its rank, and the directional edges display the largest transition probabilities for each category. The size of each node is proportional to its degree. Several notable nodes stand out as key attractors for other nodes, namely “Soft drink,” “Onigiri/Bento,” “Beer,” and “Side dishes”. The most popular categories “Soft drink” and “Onigiri/Bento” display the highest degree, due to their popularity these categories act as large attractors for other less popular categories. But interestingly there are categories whose relationships exerts a stronger influence, surpassing the attractive force of the strongly attracting categories. These findings have significant implications for understanding consumer behaviour and category associations. While popular categories undoubtedly have strong influences, it is equally essential to consider the network dynamics and the strength of connections between less popular categories that might reveal subnetworks as can be seen with “Beer” and “Side dishes” in Fig. 7c.

#### Stopping probability

The choice of stopping probability is of paramount importance to the model, as it directly controls the size of the purchases generated. Since different categories are purchased in varying quantities, it was essential to introduce a state-dependent stopping probability based on data. To achieve this, we introduced the concept of conditioned mean purchase size, denoted as $\textrm{E}(n|c)$. This quantity represents the expected purchase size *n* conditional on the purchase containing category *c*. To better describe this quantity, let us first define the subset $V = \{ n \mid \text {purchase of size } n \text { contains category } c \}$, which in turn define the conditioned mean purchase size as

$$\begin{aligned} \textrm{E}(n|c) = \frac{1}{\left| V \right| } \sum _{n \in V} n \;, \forall c \in C \end{aligned}$$

Then utilising the conditioned mean purchase size, the stopping probability is approximated using

2 $$\begin{aligned} P_S(c) = \frac{k_s}{\textrm{E}(n|c)} \;, \forall c \in C \end{aligned}$$

The Markov model that uses this stopping probability we refer to as the **simple Markov model**. Here, $k_S$ serves as a hyperparameter, allowing us to tailor the model to fit the real data. While the model currently operates as a true Markovian process, as shown in Fig. 1, this alone is insufficient to fully capture all the purchase behaviour observed in the POS data. This leads to the expansion of Eq. (2). Its important to note $k_s$ is conditioned $k_s \le \min (\textrm{E}(n|c))$ which ensures $0 \le P_S(c) \le 1$.

To address the limitation of the simple Markov model, we introduce the **extended Markov model**, which utilises the stopping probability

3 $$\begin{aligned} P_{S,t}(c,n_t) = \frac{k_e}{\textrm{E}(n|c) (1+ \frac{n_t}{k_0})} \;, \forall c \in C \end{aligned}$$

after a purchase size activation threshold $k_t$ is reached. Thus rendering the extended Markov model truly an extension of the simple Markov model, $k_t$ acts as a switch for when to transition to the power law behaviour which the stopping probability in Eq. (3) induces. In the extended Markov models stopping probability, $k_e$ and $k_0$ are additional hyperparameters that allow for more adjustment of the stopping probability, and $n_t$ is the purchase size at time step *t*. By considering the purchase history with the term $(1+\frac{n_t}{k_0})$, the extended model introduces a temporal element to the stopping probability, enabling a more accurate representation of real-world purchase behaviours. Thus capturing the power law tail seen in Fig. 6 when the purchase size is large. Since the stopping probability effectively acts an absorbing state within the network, the introduction of this temporal element to the stopping probability gives the model a form of memory, which renders the process non-Markovian hence the name. Similarly to the simple Markov model $P_{S,t}(c,n_t)$ is conditioned to $0 \le P_{S,t}(c,n_t) \le 1$. The choice of all hyperparameters mentioned up until this point and their chosen values are explained in the coming model fitting section.

#### Starting node scheme

One significant challenge that arose during the construction of this model was task of selecting a starting node. In real-world scenarios, customers often have a driving product that leads them to make a purchase, such as a refreshing drink on a hot day. Unfortunately, this crucial information is lost in the POS dataset, as the POS data consists of customer receipts whose products are in a non-chronological order. Thus there is no means of determining or calculating the starting probability for different categories. Regrettably, this limitation stands as one of the most significant drawbacks of the dataset. To address this issue, a scheme for selecting a starting node when iterating the network had to be devised. This led us to introduce the node count vector ${\textbf {N}}_t$, containing a value for each category in the network at time step *t*. Initially, ${\textbf {N}}_t$ starts as a uniform vector, with all elements equal to one: ${\textbf {N}}_0 = [1, 1, \dots , 1]$ for all categories $c \in C$. The starting probability for a node is determined proportionally to its node count. We calculate the starting node probabilities using

4 $$\begin{aligned} P_{I,t}(c) = \frac{{\textbf {N}}_t(c)}{\sum _{j=1}^C{\textbf {N}}_t(c)} , \forall c \in C \end{aligned}$$

**Figure 8:**
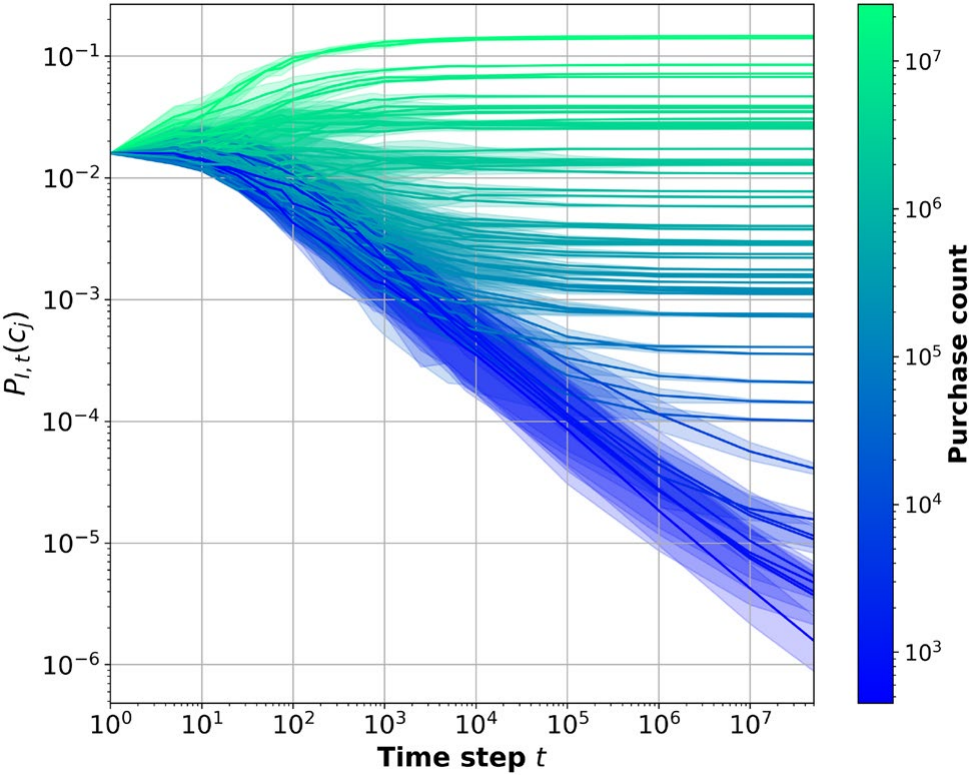
Starting node probability $P_{I,t}(c)$ convergence over time, coloured according to category purchase count from table 1. For each category the mean and 95% confidence interval is displayed.

The starting node probability $P_{I,t}(c)$ for category *c* at time step *t*, is calculated by taking the node count for category *c* at that time step and normalising it by the sum of all node counts, as seen in Eq. (4). Initially all categories have a node count of one, resulting in all nodes having a uniform probability of being selected as the starting node, which is not representative of reality. Thus we introduce the scheme which will give us an approximation of the true starting node probabilities. As the network iterates, a start node is selected, transitions between nodes occurs and eventually the process stops due to the stopping probability, resulting in one purchase. Each time a node *c* is chosen as the starting node using $P_{I,t}(c)$ or as the destination of a transition using $P_T(c)$, its respective node count ${\textbf {N}}_t(c)$ is increased by one. Consequently, as the network is iterated, ${\textbf {N}}_t$ will evolve over time, guided by the starting probabilities and transition probabilities. This adaptive approach enables the model to simulate the dynamics of customer purchase behaviour while accounting for the absence of explicit starting probabilities in the original dataset. Interestingly it was found that the starting node probability converged over time which can be seen in Fig. 8. These results show the mean and standard deviation from 10 runs of the simulation where $5 \times 10^7$ purchases where generated, reflecting the number of purchases in the POS data. Notably, the starting probabilities $P_{I,t}(c)$ gradually stabilise over time for most categories especially the more popular categories. As observed in Fig. 8 the less popular categories take longer to converge, some of the least popular categories does not converge at all, reflecting their infrequent purchase occurrences. Given the observation that $P_{I,t}(c)$ converges over time for the majority of categories, and under the assumption that the least popular categories are close to converging at $5*10^7$ simulated purchases. We now eliminate its time dependency and instead consider the stationary starting probabilities denoted as $P_{I}(c)$. The resulting converged starting probabilities serve as a fundamental aspect of the model.

**Figure 9:**
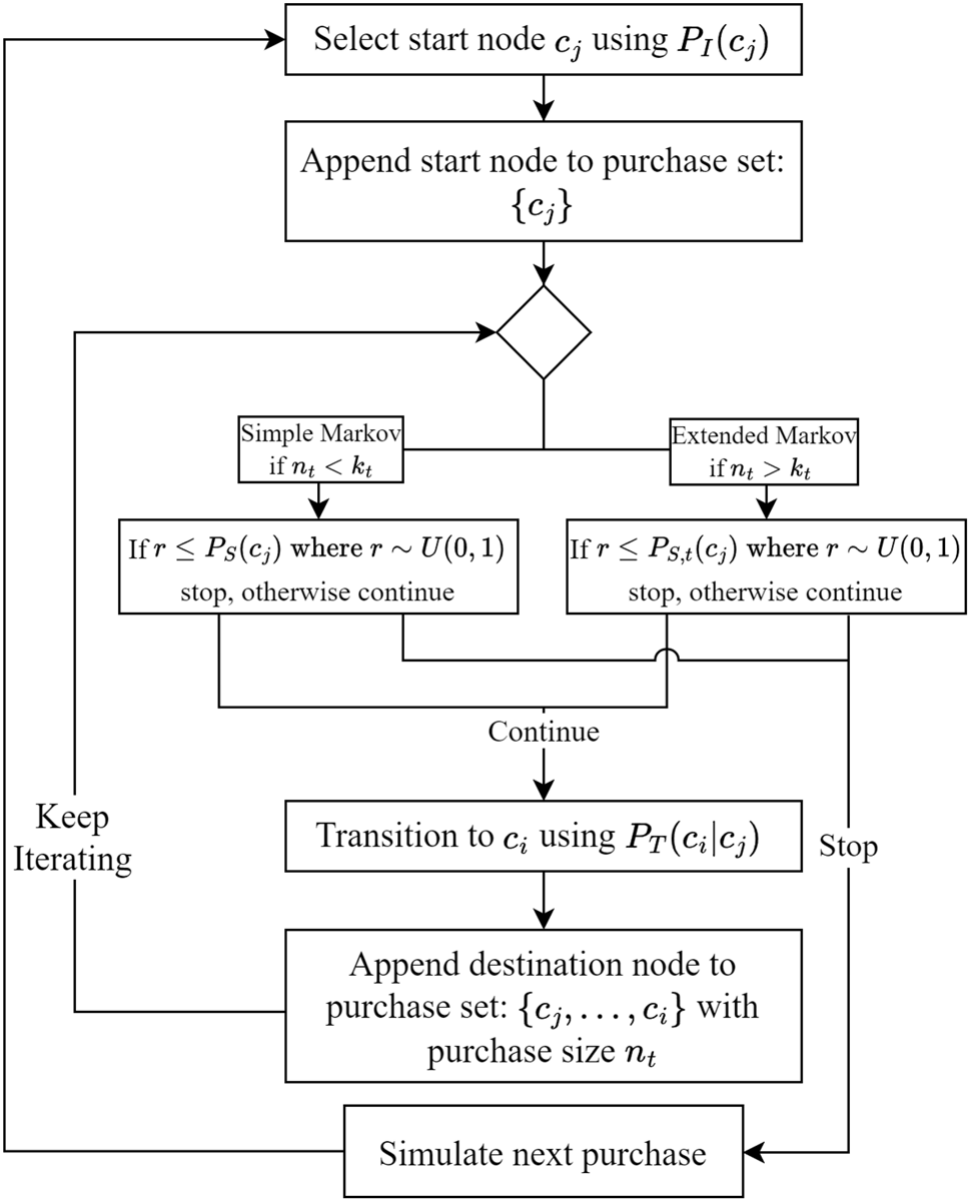
Flowchart of model showing the process of generating purchases.

**Figure 10:**
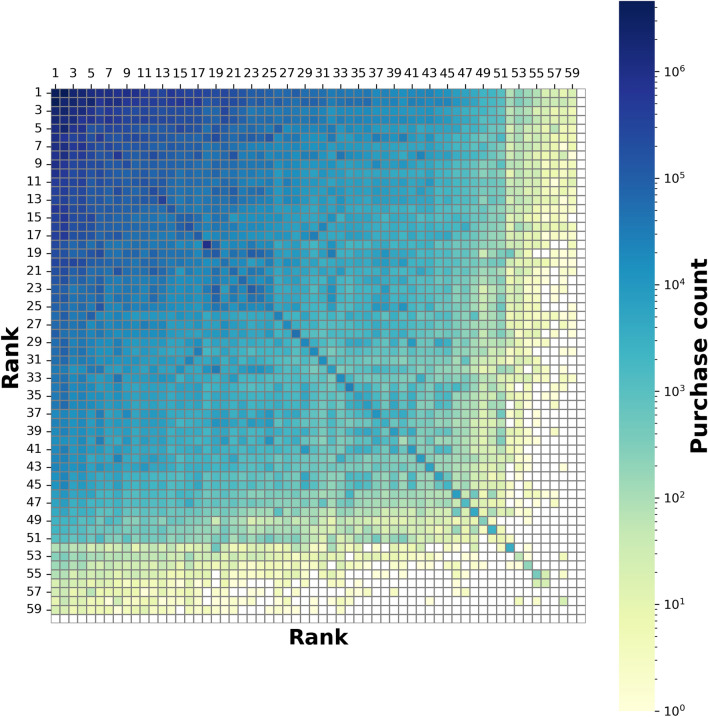
Heat map of the category matrix showing all categories according to their rank and colored by the category combination purchase count.

Now, given all the individual components of the Markov models, Fig. 9 depicts a flowchart for the generation of synthetic purchases. Using the presented methodology, all purchases analysed in the result section were synthesised.

#### Model fitting

For optimal performance of the models presented, it was imperative to fine-tune their hyperparameters. This tuning was achieved through a traditional grid search approach. The performance metric used for this optimisation was the root mean squared error between the predicted and actual data, specifically in the CDF space, as illustrated in Fig. 1. For the simple Markov model $k_s = 1.42$ was found to have the best fit. For the extended model below activation threshold for the extended stopping probability $k_s = 1.42$ was also used. The activation threshold $k_t$ and $k_e, k_0$ were fitted in the same fashion, their values was found to be $k_t = 18$, $k_e = 2.15$ and $k_0 = 10$. Using the these hyper parameters, all purchases analysed in the result section were synthesised.

### Driving force

Driving force refers to the influence of purchasing product *j* on the likelihood of subsequently purchasing product *i*, as opposed to making random purchases. This metric measures the inclination to buy more items from category *i* after already choosing a product from category *j*. The driving force present in the real and simulated purchases is thus defined as

5 $$\begin{aligned} D_{i,j} = \frac{\textrm{E}(n_i|c_j)_{\textrm{m}}}{\textrm{E}(n_i|c_j)_{\textrm{Null}}}, \; m = \textrm{Real, Simulated} \end{aligned}$$

which calculates the driving force for the target category *i* conditioned on category *j*. Here, $\textrm{E}(n_i|c_j)$ represents the mean number of category *i* in a purchase, conditioned on the presence of category *j* in that same purchase.

In simpler terms, $\textrm{E}(n_i|c_j)$ measures how much of category *i* is typically bought when category *j* is present in the purchase. The driving force is then computed as the ratio of these conditional means between the real data and the random purchase data from the null model, as shown in Eq. (5). A description of the null model is provided in the following section. The driving force provides valuable insights into the magnitude of the purchase driving forces that exists between different product categories in the real or simulated data, relative to random purchases. If the ratio is greater than one, it indicates that the presence of category *j* positively influences the likelihood of purchasing category *i* beyond what would be expected by random chance. By examining these driving force ratios for various category pairs, meaningful relationships between product categories can be identified. For instance, a ratio significantly greater than one suggests a strong association, where the presence of one category consistently drives the purchase of another.

#### Null model

The null model consists of randomised purchase data, which was generated using a straightforward randomisation scheme. Initially, all real purchases are converted into empty sets, while still retaining their original purchase sizes. Subsequently, all the products from these purchases were combined into a large vector ${\textbf {G}}$. To create the randomised purchases, we randomly sampled from ${\textbf {G}}$, without replacement and filled the empty purchase sets with these randomly selected products. This randomisation process guarantees that the inter-category relationships are lost to randomness, effectively breaking any existing associations between products. However, it ensures that the overall purchase size distribution and the ratio of product categories are preserved in the randomised data.

Using the above method many new sets of randomised purchases were generated. The null model was then created by averaging $\textrm{E}(n_i|c_j)$ for all categories over all random sets, resulting in $E(n_i|c_j)_{Null}$.

## Data Availability

The data was provided by Seven-Eleven Japan Co., Ltd. for academic study in 2010. The raw data cannot be copied freely without permission of the data provider, Seven-Eleven Japan Co., Ltd. How to access the data: POS data in general can be purchased from data provider companies or be obtained directly from individual companies which are using POS data in their business. To request the data used in this study, contact Misako Takayasu^∗^.

## References

[1] SEVEN-ELEVEN JAPAN CO., L. Corporate profile. https://www.sej.co.jp/company/en/c_profile.html (2023).

[2] Marchuk Y (2018). Predicting patient-ventilator asynchronies with hidden Markov models. Sci. Rep..

[3] Foucrier A (2022). Transition matrices model as a way to better understand and predict intra-hospital pathways of covid-19 patients. Sci. Rep..

[4] Cruz-Monteagudo M (2007). Computational chemistry development of a unified free energy Markov model for the distribution of 1300 chemicals to 38 different environmental or biological systems. J. Comput. Chem..

[5] Nguyen N, Nguyen D (2021). Global stock selection with hidden Markov model. Risks.

[6] Liu L, Liu J, Zhou Q (2022). Mine ventilation system reliability evaluation based on a Markov chain. Sci. Rep..

[7] Ma R, Zheng X, Wang P, Liu H, Zhang C (2021). The prediction and analysis of COVID-19 epidemic trend by combining LSTM and Markov method. Sci. Rep..

[8] Chintagunta PK, Haldar S (1998). Investigating purchase timing behavior in two related product categories. J. Mark. Res..

[9] Young MR, Desarbo WS, Morwitz VG (1998). The stochastic modeling of purchase intentions and behavior. Manag. Sci..

[10] Lu X, Wetter E, Bharti N, Tatem AJ, Bengtsson L (2013). Approaching the limit of predictability in human mobility. Sci. Rep..

[11] Awad MA, Khalil I (2012). Prediction of user’s web-browsing behavior: Application of Markov model. IEEE Trans. Syst. Man Cybern. B (Cybern.).

[12] Cui Z, Lin L, Pu Z, Wang Y (2020). Graph Markov network for traffic forecasting with missing data. Transp. Res. C: Emerg. Technol..

[13] Meng, X., Lee, K. K. & Xu, Y. Human driving behavior recognition based on hidden markov models, in *2006 IEEE International Conference on Robotics and Biomimetics*, 274–279, 10.1109/ROBIO.2006.340166 (2006).

[14] Jandera A, Skovranek T (2022). Customer behavior hidden Markov model. Mathematics.

[15] Sakoda G, Takayasu H, Takayasu M (2020). Metabolic dynamics of ecosystems realizing steady log-uniform distributions: The case of commodities in shops. Entropy.

[16] Sakoda G, Takayasu H, Takayasu M (2019). Data science solutions for retail strategy to reduce waste keeping high profit. Sustainability.

[17] Sakoda G, Takayasu H, Takayasu M (2019). Tracking Oisson parameter for non-stationary discontinuous time series with Taylor’s abnormal fluctuation scaling. Stats.

[18] Fukunaga G, Takayasu H, Takayasu M (2016). Property of fluctuations of sales quantities by product category in convenience stores. PLoS ONE.

